# A comprehensive analysis of the prognostic value, expression characteristics and immune correlation of MKI67 in cancers

**DOI:** 10.3389/fimmu.2025.1531708

**Published:** 2025-02-24

**Authors:** Xiaolan Pan, Caibiao Wei, Jingyu Su, Min Fang, Qiumei Lin, Yuling Qin, Jie Gao, Jie Zhao, Huiliu Zhao, Fengfei Liu

**Affiliations:** ^1^ Department of Clinical Laboratory, Guangxi Medical University Cancer Hospital, Nanning, China; ^2^ Genetic Metabolism Center laboratory, Guangxi Zhuang Autonomous Region Maternal and Child Health Care Hospital, Nanning, China; ^3^ Department of Medical Records, Guangxi Medical University Cancer Hospital, Nanning, China

**Keywords:** MKI67, prognosis, cancer immunity, pathway, multi-omics bioinformatics

## Abstract

**Background:**

nuclear-associated antigen Ki67 (Ki67) emerges as a clinically practical biomarker for proliferation assessment among many cancer types. However, the definite prognostic value of Ki67 against a specific cancer type has remained vague. This study aims to perform a comprehensive pan-cancer analysis of the prognosis value of Ki67 across various cancer types.

**Methods:**

This study explored the expression, prognostic value, and tumor-infiltrating immune of MKI67 in the TCGA database by pan-cancer, and then performed immunohistochemical, correlation analysis and prognostic analysis using 10028 patients of the top 10 cancer patients in China we collected. The correlation between MKI67 expression and survival outcome, clinical features, MSI, TMB, and tumor-infiltrating immune cells by TCGA database, xCell, and TIMER algorithms.

**Results:**

MKI67 expression was significantly upregulated across varied cancer types verified by datasets. We found MKI67 expression was significantly associated with poor prognosis in LUADLUSC, LIHC, and BRCA patients, but good prognosis in COADREAD and READ patients via Kaplan-Meier survival analysis using 10028 patients collected. These results of our validation were generally consistent with TCGA database except BRCA, COADREAD and READ. Meanwhile, upregulation of MKI67 elevates the degree of immune infiltration of several immune cell subtypes, such as functional T cells, CD4^+^ T cells, and CD8^+^ T cells, as well as, MKI67 was related to Cell cycle, Oocyte meiosis, p53 and other pathways.

**Conclusion:**

Our comprehensive analysis may supply useful guidance on MKI67 applicability across various cancer types. These observed results contribute to the promise of MKI67 in a realistic clinical setting and improve the outcomes of cancer patients.

## 1 Introduction

Cancer prognosis is involved in hallmark histopathological, immune infiltration, genomic, and transcriptomic heterogeneity of the tumor and tissue microenvironment, giving rise to varied treatment response rates and patient outcomes (1–4). The clinical settings of multiple cancer types exert several parameters such as tumor, nodes, and metastases (TNM) staging system, pathological type, clinical stage, and Nuclear-associated antigen ki-67 (Ki67) scores to divide patients into different risk groups for diverse therapeutic patterns (5–7). Notably, it is widely acknowledged that Ki-67 emerges as a clinically practical biomarker for proliferation assessment among many cancer types (8, 9). Ki67 is a macromolecular protein encoded by MKI67 gene and expressed in the nucleus, which is a common indicator for detecting cell proliferation activity (10, 11). The expression of Ki67 protein is related to the proliferative activity of endogenous cell population in malignant tumors, so it can be used as a reference index of tumor invasiveness and plays an important role in the grading of malignant tumors (12–14). Meanwhile, the prognostic evaluation value of Ki67 has been carried out in some studies, and it can be used as a reliable marker to evaluate the prognosis of breast cancer, lung cancer(LUADLUSC), and cervical cancer, etc. (15–18). For example, Spratt et al. analyzed the studies on Ki67 and prostate tumors and found that high expression of Ki67 was closely related to poor prognosis of prostate tumors (17). And overexpression of Ki67 in patients with gastric cancer may lead to disease progression and metastasis, and may affect the metastasis of gastric tumors to lymph nodes (19, 20). What’s more, MKI67 plays a crucial role in promoting T cell depletion within Liver hepatocellular carcinoma (LIHC) (21–23). These findings suggest that MKI67 may play different regulatory roles in the progression of human cancers, which may include the regulation of tumor cell proliferation, migration, and the tumor immune microenvironment. Additionally, Ki67 is overexpressed in autoimmune diseases such as psoriasis and rheumatoid arthritis due to the roles of proliferation regulation (24–27). However, despite the plentiful studies examining Ki67 immunohistochemical analysis among multiple cancer types, its adoption into clinical practice has been limited to different regulatory roles, different prognosis values, and varied cutoff values against certain cancer types (28–30).

Recent advancements achieved in the clinical application of Ki67, such as different scoring methods of Ki67, definitive prognosis value of certain cancer types, and decisive cutoff values for specific cancer type or subtype (29, 31–33). For instance, the low expression group of Ki67 had a longer OS than the high one in LUADLUSC, while the increased expression of MKI67 had a longer OS in Colorectal carcinoma(COADREAD) (34). Additionally, some studies reported that Ki67 had no prognostic value in gastric cancer, and may only be a potential indicator of intra-tumor heterogeneity. A study reported that a cutoff value of MKI67 at 55% was capable of dividing G3 neuroendocrine neoplasms (NENs) into two different prognostic groups (35, 36). However, there has not been a comprehensive analysis of Ki67 among numerous cancer types to date, making Ki67 of prognosis utility in a certain cancer type remaining vague.

In this study, we firstly identified the definite prognosis value of the top 10 cancer types in China (37) using 10028 patients collected, i.e., LUADLUSC, COADREAD, Thyroid carcinoma(THCA), LIHC, Stomach adenocarcinoma (STAD), Breast invasive carcinoma(BRCA), Esophagus squamous cell carcinoma(ESCA), Cervical endocervical adenocarcinoma and squamous cell carcinoma(CESC), Prostate adenocarcinoma(PRAD) and Pancreatic adenocarcinoma (PAAD), which were investigated the differential expression levels of MKI67 in cancer and normal tissues by several public databases. Moreover, we thoroughly revealed the correlation between MKI67 and tumor-infiltrating immune cells and the related pathways using pan-cancer datasets. We further verified the correlation between the Ki67 stratification and clinicopathological stage through 10028 patients collected. We aim to supply a comprehensive analysis of the prognosis value of Ki67 among various cancer types.

## 2 Materials and methods

### 2.1 Clinical data collection

**Figure 1:**
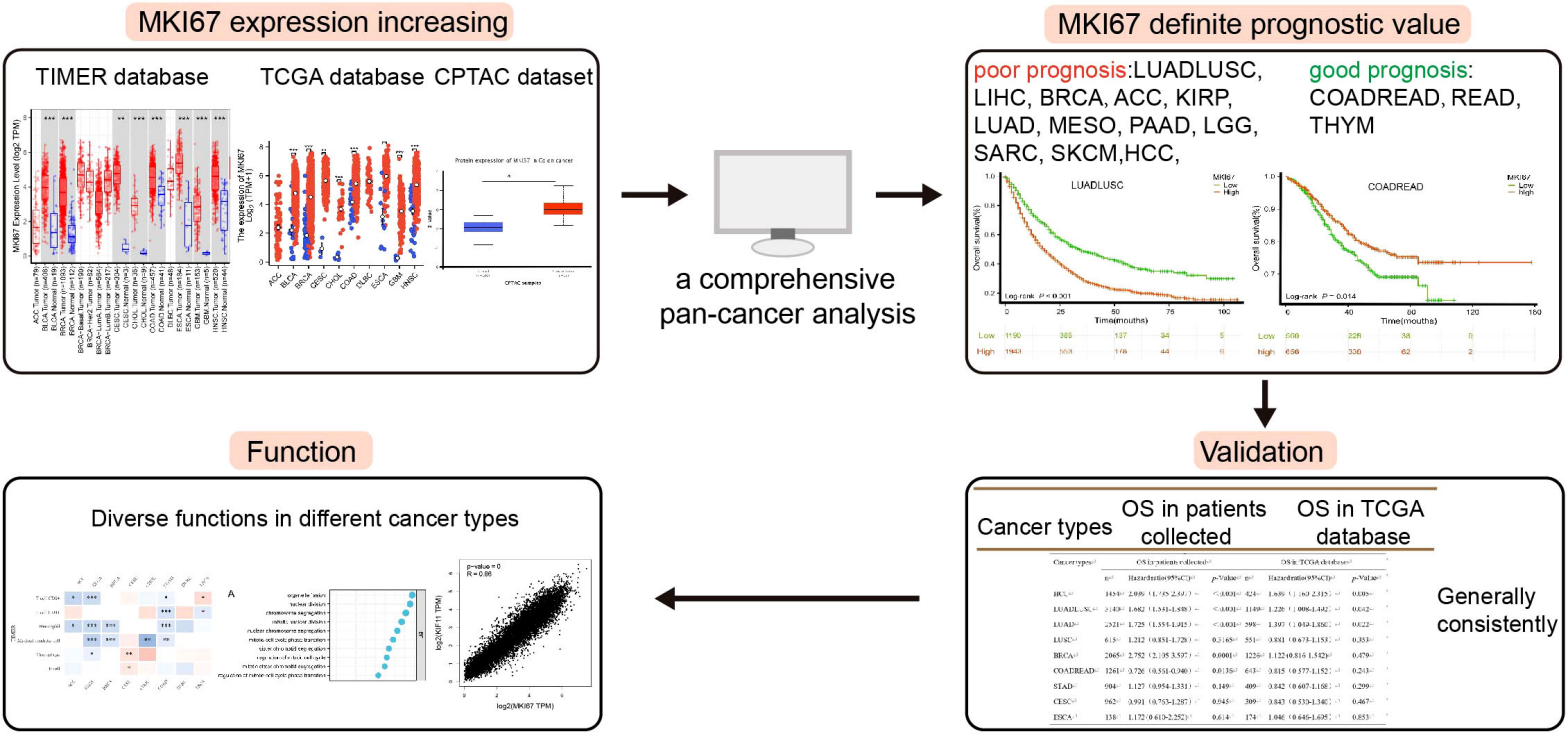
A workflow diagram of this study.

Clinical and pathological data of 55,230 cancer patients admitted to Guangxi Medical University Cancer Hospital (Nanning, China) from January 2013 to October 2022 were recorded. The criteria for inclusion in this study were as follows: (I) pathologically confirmed LUADLUSC, COADREAD, THCA, LIHC, STAD, BRCA, ESCA, CESC, PRAD and PAAD; (II) primary tumor resection carried out postoperative gross specimen analysis with detecting Ki67. (III) the absence of prior anticancer treatment; (IV) the absence of concurrent malignancies; (V) availability of comprehensive laboratory, pathological, and follow-up data. The exclusion criteria were: (I) preoperative treatment (including radiation, chemotherapy, or chemoradiotherapy); (II) exposure to other types of cancer before or after diagnosis; (III) known familial history of cancer; (IV) unavailability of comprehensive laboratory, pathological, and follow-up data. According to the inclusion and exclusion criteria, 10028 cancer patients (1261 patients with COADREAD, 904 patients with STAD, 1454 patients with LIHC, 2065 patients with BRCA, 138 patients with ESCA, 926 patients with CESC, 46 patients with PRAD and 58 patients with PAAD) participated in the study. At the same time, relevant information such as age, sex, pathological stage and prognosis information were collected. The protocol of this study was approved by the Ethics and Human Discipline Committee of Guangxi Medical University Cancer Hospital (LW2024033), and all experiments and methods conformed to the standards of relevant guidelines and regulations. And the specific research process was shown in **Figure 1**.

### 2.2 Gene expression analysis

TIMER2 (Tumor Immune Estimation Resource, version 2, http://timer.cistrome.org/) was used to analyze the expression profile of MKI67 between tumor tissues and adjacent normal tissues, and the gene expression levels are represented as log2 TPM values. The MKI67 mRNA expression profiles and correlative clinical data from 33 types of cancer samples and corresponding normal samples were downloaded from TCGA (https://portal.gdc.cancer.gov). There are fewer than 3 samples in the current data group or 0 standard deviations (SD) within the group (SARC, SKCM, THYM, ACC, DLBC, LAML, LGG, MESO, OV, SKCM, UCS, UVM), these groups will not be included for statistical analysis (visualization will still be performed). The Clinical Proteomic Tumor Analysis Consortium (CPTAC) database (https://cptac-data-portal.georgetown.edu/) was used to investigate the MKI67 protein expression level in normal tissues and primary tissues.

### 2.3 Pathologic staging analysis

We extracted the expression data of MKI67 gene in each sample from the TCGA database, and further screened the samples from Primary Blood Derived Cancer-Peripheral Blood Primary Tumor. Log^2(x+0.001)^ transformation was performed for each expression value, and cancer species with less than 3 samples in a single cancer species were eliminated. R software (version 4.0.3) was used to calculate the expression difference of genes in different clinical stage samples in each tumor. Using unpaired t-tests for two-by-two differences and ANOVA for differences in multiple groups of samples.

### 2.4 Immunohistochemistry staining and evaluation

Paraffin-embedded tissue samples from LUADLUSC, COADREAD, LIHC, STAD, BRCA, ESCA, CESC patients were skillfully sectioned into slices measuring 4 μm in thickness. These sections were dewaxed using xylene and subsequently rehydrated through a series of alcohol washes. To ensure optimal quality, all sections were repaired by microwave heating while endogenous peroxidase activity was inhibited using a 3% H_2_O_2_ solution. Following these preparatory steps, the sections were subjected to overnight incubation at a temperature of 4°C utilizing an anti-Ki-67 antibody (dilution 1:150; Maixim, Kit-0030). The subsequent immunohistochemical analysis was executed using the DAKO EnVision detection system.

To ensure unbiased evaluation, two independent pathologists carefully evaluated immunohistochemical staining scores for Ki-67 in tissues without knowledge of relevant clinical data. This evaluation was performed by semi-quantitative methods. Staining scores were categorized into four different levels:0 (negative), 1 (weak), 2 (moderate), and 3 (strong). Specifically, high expression was defined as a staining score of more than 2, in which at least 75% of the malignant cells exhibited positive staining. In contrast, moderate expression was defined as a staining score of 2 accompanied by at least 25% of malignant cells showing positive staining. Finally, low expression implied a staining score of less than 2, indicating that less than 25% of malignant cells showed positive staining.

### 2.5 Survival prognosis analysis

Survival and clinical phenotype data were extracted from each sample downloaded from TCGA. Overall survival (OS), disease-specific survival (DSS), disease-free interval (DFI) and progression-free interval (PFI) were selected to investigate the relationship between MKI67 expression and patient prognosis. Kaplan-Meier method and log-rank test were used for survival analysis for each cancer type (*p* < 0.05). Survival curves were plotted using the R package “survival” and “survminer”. In addition, Cox analysis was performed using the R package “survival” and “forest plot” to determine the pan-cancer relationship between MKI67 expression and survival.

### 2.6 Follow-up for survival

Patient follow-up was assiduously managed by professionals, using telephone contacts or outpatient monitoring systems to determine the patient’s illness status or date of death. OS was defined as the time between surgery and patient death or the end of follow-up. After surgery, patients were monitored systematically at specified intervals. The calculation of OS requires determining the span of time between the patient’s date of resection and the date of death or last follow-up, with October 31, 2022 as the end date. We used a full case analysis (CCA) to address potential biases or challenges encountered during follow-up (38).

### 2.7 Immunoinfiltration analysis

RNAseq data (level 3) and corresponding clinical information for pan-cancer were obtained from the TCGA database. To perform a reliable assessment of immune relevance, we used immunedeconv, an R package that integrates two of the latest algorithms, including TIMER and xCell. The expression values of 8 genes were extracted to observe the expression of immune checkpoint related genes. These 8 genes, including SIGLEC15, IDO1, CD274, HAVCR2, PDCD1, CTLA4, LAG3 and PDCD1LG2, are the transcripts associated with immune checkpoint. TMB and MSI scores from TCGA. The correlation between MKI67 expression and TMB or MSI was analyzed by Spearman method. Statistical analysis was performed using R software v4.0.3.

### 2.8 Gene set enrichment analysis

The protein-protein interaction network was analyzed using the STRING database (https://string-db.org/). Based on TCGA database, GEPIA2 database (http://gepia2.cancerpku.cn) was used, which won the first 100 MKI67 related genes. We then performed pared gene Pearson correlation analysis for MKI67 and the first 10 genes. The biological and molecular function of MKI67 in pan-cancer was analyzed using GO (Gene Ontology) and KEGG (Kyoto Encyclopedia of Genes and Genomes) analysis, and the enrichment pathway was performed and visualized using the R-package ClusterProfiler.

### 2.9 Statistical analysis

SPSS version 26.0 software was used for processing. Pearson chi-square test was used to analyze the relationship between MKI67 expression and clinicopathological characteristics. Kaplan-Meier analysis method and Log-rank test were used for survival analysis. Differences between groups were analyzed using the Student’s t-test. *p* < 0.05 (two-tailed) was considered statistically significant.

## 3 Results

### 3.1 MKI67 expression in human cancer types

**Figure 2:**
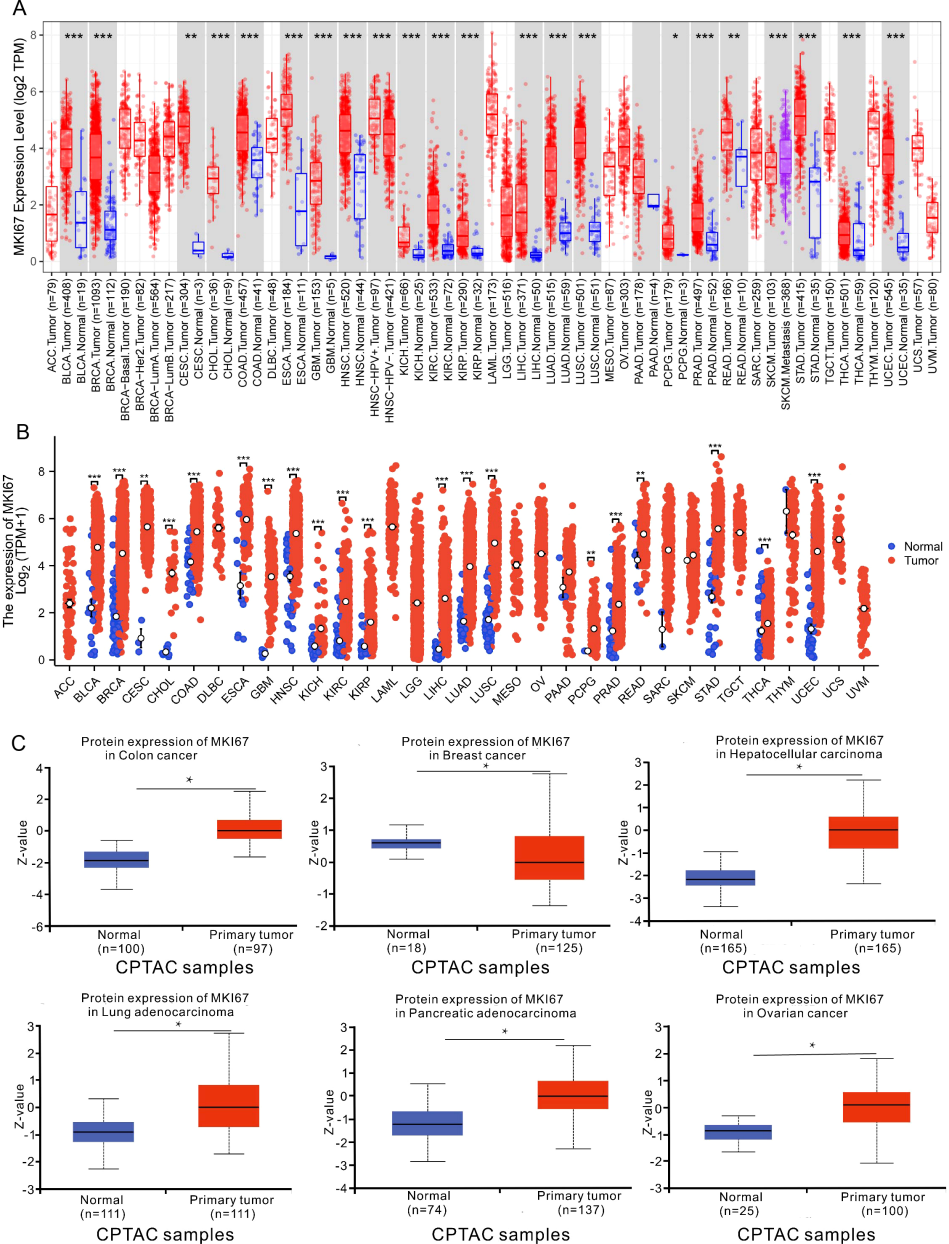
Upregulated mRNA and protein expression of MKI67 in pan-cancer. **(A)** The results from the TIMER database indicated that the MKI67 expression was remarkably increased in 16 cancer types. The red and blue boxes represent tumor tissues and normal tissues, respectively. **(B)** The expression level of MKI67 in different cancer types from TCGA. **(C)** The MKI67 protein expression level in normal tissues and primary tissues in the CPTAC dataset. ∗*p* < 0.05, ∗∗*p* < 0.01, and ∗∗∗*p* < 0.001.

We identified the MKI67 mRNA expression characteristics between pan-cancer and adjacent normal tissues using the TIMER2 database and TCGA data. As shown in **Figure 2A**, MKI67 was upregulated across diverse cancer types, including BLCA, BRCA, CESC, CHOL, COAD, ESCA, GBM, HNSC, KICH, KIRC, KIRP, LIHC, LUAD, LUSC, PCPG, PRAD, READ, SKCM, STAD, THCA and UCEC. **Figure 2B** exhibited that the MKI67 expression was upregulated among diverse cancer types consistent with the results of the TIMER2 dataset. We further evaluated MKI67 protein levels between pan-cancer and adjacent normal tissues using the CPTAC dataset in transcriptional levels. **Figure 2C** indicated MKI67 protein expression was significantly higher among COAD, BRCA, LIHC, LUAD, PAAD, and OV consistent with TIMER2 database and TCGA data. Overall, MKI67 expression was significantly upregulated across varied cancer types.

### 3.2 Pan-cancer analysis of the correlation between MKI67 expression and clinicopathology

**Figure 3:**
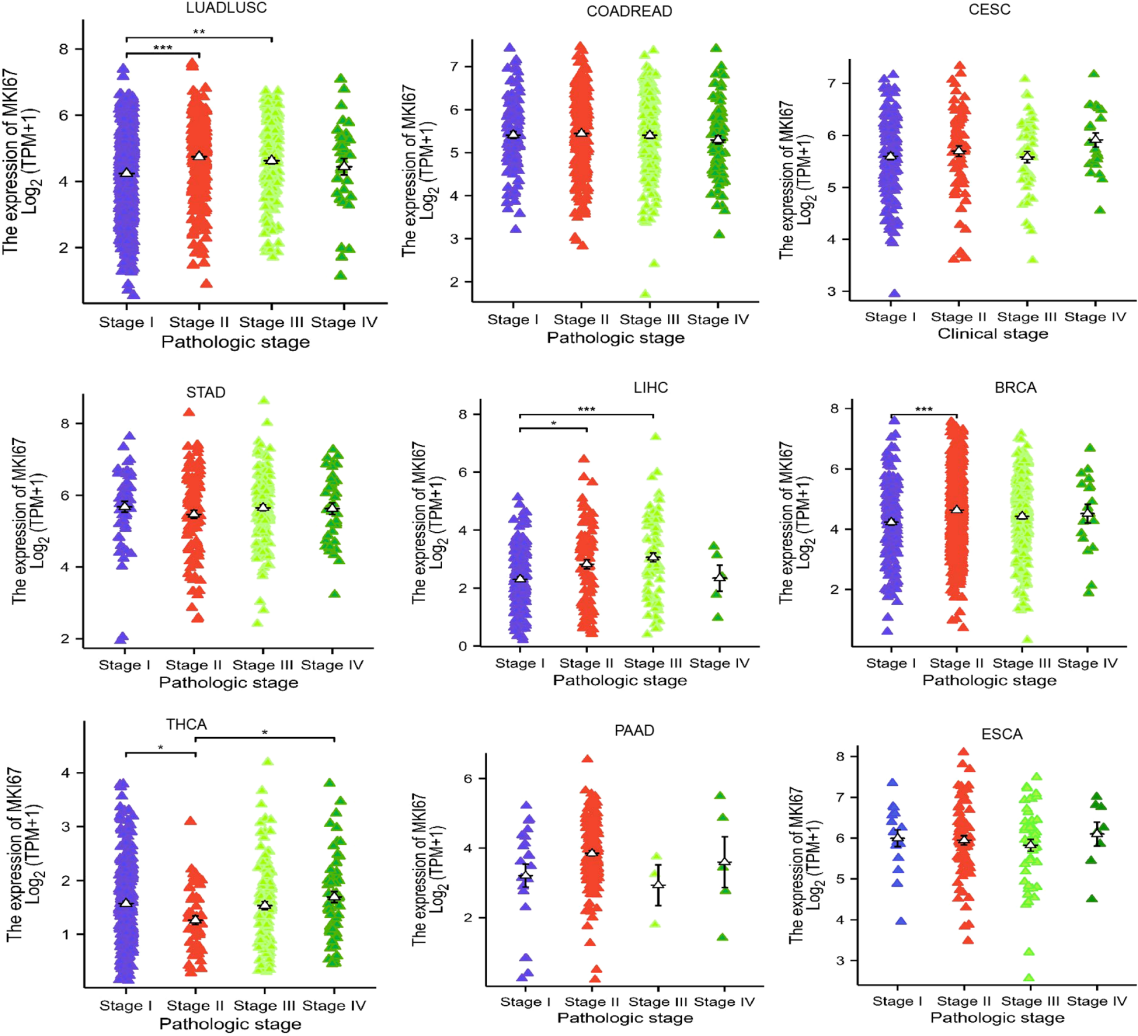
Correlations between the MKI67 expression and the main pathological stages, including stage I, stage II, stage III, and stage IV of LUADLUSC、COADREAD、CESC、STAD、LIHC、BRCA、THCA、PAAD and ESCA, were investigated based on the TCGA data. Log^2 (TPM+1)^ was used for log scale. ∗*p* < 0.05, ∗∗*p* < 0.01, and ∗∗∗*p* < 0.001.

After identifying the characteristics of MKI67 expression at the mRNA and protein levels, we explored the association between MKI67 expression and clinicopathological features and clinical parameters across different cancer types using the TCGA database and the 10028 cancer patients data we collected, respectively. **Figure 3** revealed that there were significantly different MKI67 expressions of stage I, II, III, and IV among LUADLUSC, LIHC, BRCA, and THCA based on the TCGA database. Additionally, there were not statistical different MKI67 expressions of stage I, II, III, and IV among COADREAD, CESC, STAD, PAAD, and ESCA in the TCGA database. We then probed the correlation of MKI67 expression with clinical parameters in 10028 cancer patients collected including LUADLUSC, COADREAD, THCA, LIHC, STAD, BRCA, ESCA, CESC, PRAD and PAAD. **Supplementary Table S1** shown poor differentiation, TNM classification, classification, and staging in BRCA patients were correlated with high expression of MKI67. **Supplementary Table S2** indicated the high expression of Ki67 was closely related to TNM classification, clinical stage and pathological type in LUADLUSC patients. **Supplementary Table S3** indicated the BCLA stage, Edmondson grade, tumor size and tumor nodules increasing in LIHC patients were closely related to the high expression of Ki67. **Supplementary Table S4** displayed the high expression of Ki67 was associated with N classification, M classification, pathological type, and differentiation degree in COADREAD patients. However, **Supplementary Tables S5**–**S7** showed that the expression of MKI67 was unrelated to the clinicopathology of STAD, ESCA, and CESC. Whereas, the clinical information of CESC, STAD, and ESCA was too little to be analyzed, and the amount of data of THCA, PRAD and PAAD were too small to be validated. Above all, the results of TCGA database and our validation present that the high MKI67 expression was associated with certain clinicopathological features and clinical parameters, for instance, poor differentiation, TNM classification, and clinical stage, among some cancer types.

### 3.3 Prognostic value of MKI67 expression in our validation cohort and TCGA database by pan-cancer analysis

**Figure 4:**
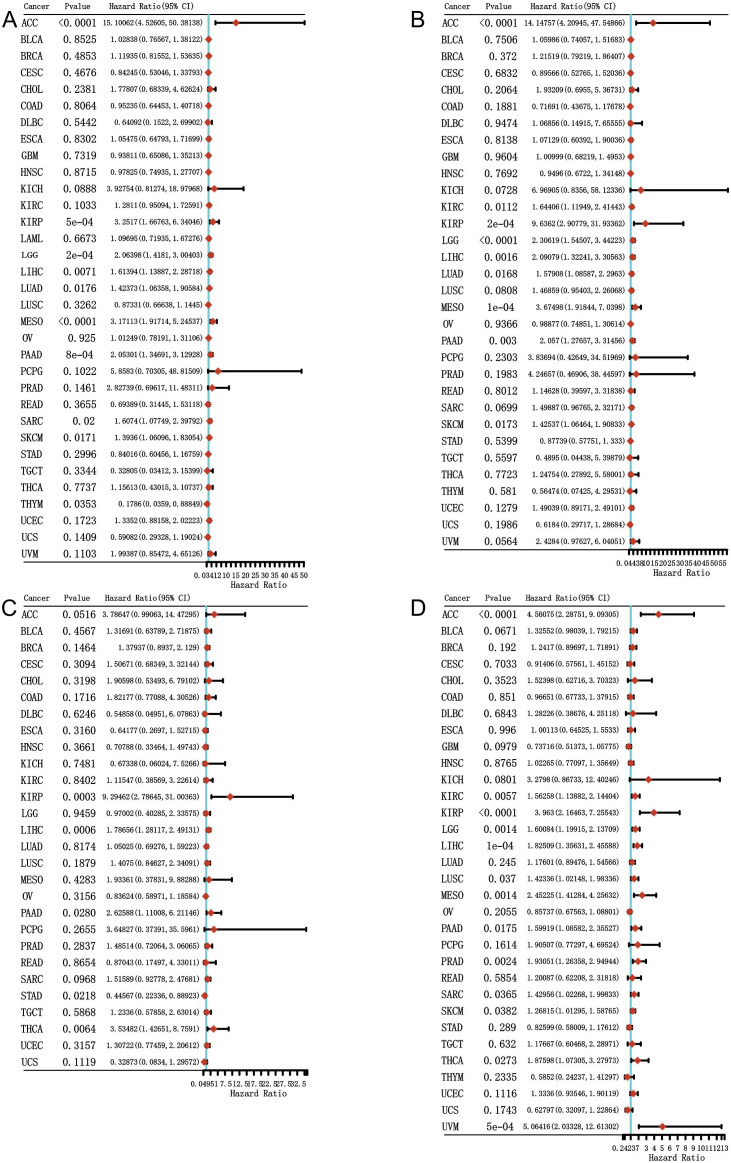
Correlation between MKI67 gene expression and survival of 33 different cancer types in TCGA database. The “survival” and “ggplot2” packages of R software were used to perform survival analyses regarding MKI67 across 33 different types of tumors. **(A–D)** The forest plots of univariate Cox regression of OS, DSS, DFS, PFS, sequentially.

To investigate the prognosis value of MKI67 expression among various cancer types, we then explored the correlation between MKI67 expression and prognosis of patients within different cancer types based on the TCGA database. We used several survival metrics including OS, DSS, DFS, and PFS to evaluate the prognosis value of MKI67 expression by Cox regression analysis. As shown in **Figure 4A**, MKI67 expression was significantly associated with OS in 10 cancer types, including ACC, KIRP, LGG, LIHC, LUAD, MESO, PAAD, SARC, SKCM, and THYM. MKI67 was a risk factor in these cancer types except THYM. **Figure 4B** displayed MKI67 expression was remarkably related to DSS of 9 cancer types, including ACC, KIRC, KIRP, LGG, LIHC, LUAD, MESO, PAAD, and SKCM. **Figure 4C** indicated that increased MKI67 expression was significantly associated with KIRP, LIHC, PAAD, STAD, and THCA of DFS. Notably, MKI67 was a risk factor for death in patients with KIRP, LIHC, PAAD and THCA but a protective factor for STAD. **Figure 4D** demonstrated MKI67 expression appreciably affected PFS in patients with KIRP, LIHC, PAAD, STAD, and THCA. Similarly, MKI67 was a protective factor for STAD but a risk factor for the others.

**Figure 5:**
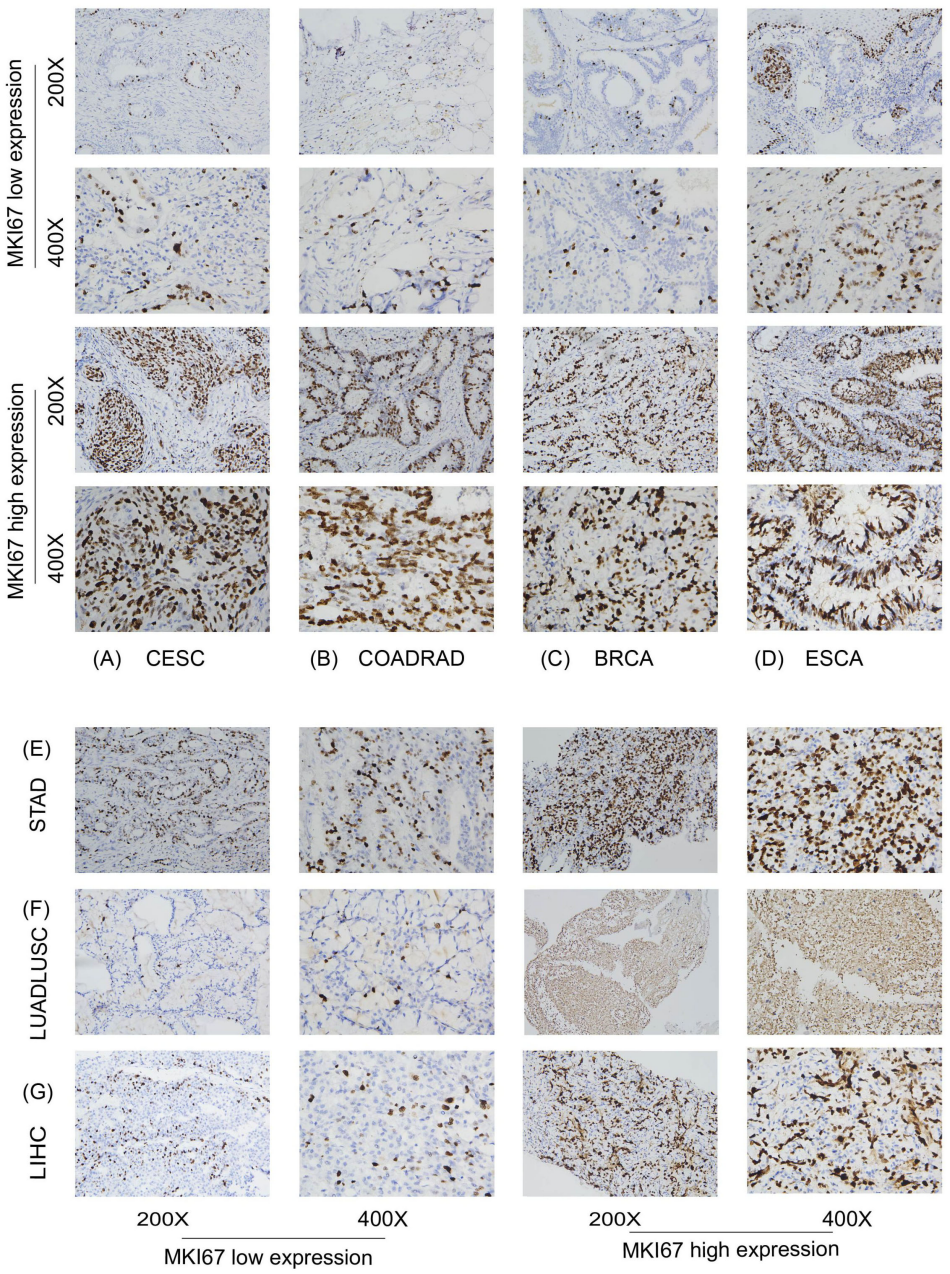
Protein expression level of MKI67 in human multiple cancer tissues of COADREAD **(A)**, BRCA **(B)**, STAD **(C)**, CESC **(D)**, LIHC **(E)**, LUADLUSC **(F)** and ESCA **(G)**. Representative images of MKI67 expression in pan-cancer tissues are shown. Original magnification, ×200 and ×400.

We further focused on the relationship between MKI67 expression and OS using Kaplan-Meier analysis based on the 10028 cancer patients collected and TCGA database. We used ROC curve to determine the cut-off value of several cancer types based on the results of IHC, to divide patients into low and high Ki67 expression groups. According to the maximized Youden Index indexes, we identified the cut-off value among different cancer types, including LUADLUSC, LIHC, BRCA, COADREAD, STAD, ESCA, and CESC, with an optimal cut-off value of 21.5%, 31.5%, 36.5%, 66.5%, 69%, 67.5%, and 74%, sequentially. **Figure 5** displayed typical IHC landscapes of low and high Ki67 expression groups in several cancer types. Based on the top 10 cancers in China, i.e. LUADLUSC, COADREAD, THCA, LIHC, STAD, BRCA, ESCA, CESC, PRAD and PAAD, we probed the association between MKI67 expression and OS by the 10028 cancer patients collected. However, the analysis of THCA, PRAD and PAAD, were not available due to limited data.

**Figure 6:**
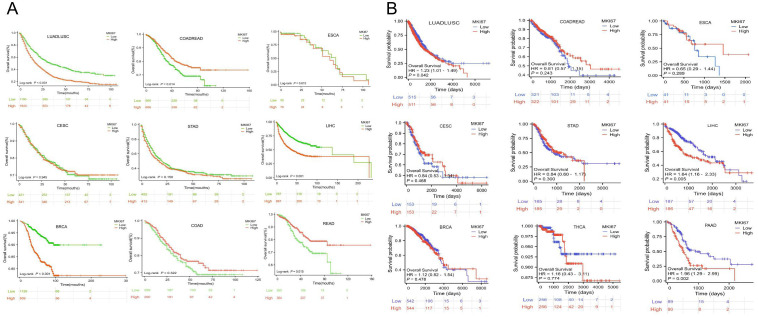
**(A)** Kaplan-Meier survival curves of OS for patients stratified by the different expressions of MKI67 in LUADLUSC、COADREAD、ESCA、CESC、STAD、LIHC、BRCA、COAD and READ by 10028 patients data. **(B)** Kaplan-Meier survival curves of OS for patients stratified by the different expressions of MKI67 in LUADLUSC、COADREAD、ESCA、CESC、STAD、LIHC、BRCA、THCA and PAAD in TCGA database.

Specially, we added the subtype of COADREAD, i.e. COAD and READ to further explore the prognosis of MKI67 expression. **Figure 6A** exhibited increased MKI67 expression was significantly associated with poor prognosis in LUADLUSC, LIHC, and BRCA patients, but good prognosis in COADREAD and READ patients by 10028 patients data. Meanwhile, we verified the association between MKI67 expression and OS using TCGA database. As shown in **Figure 6B**, the results indicated increased expression of MKI67 was notably associated with poor prognosis in patients with LUADLUSC, LIHC, and PAAD. To better compared the results of our validation and TCGA database, we used **Table 1** to clearly demonstrate the similarities and differences. **Table 1** indicated the results of our validation were generally consistent with TCGA database, except for BRCA and COADREAD. **Table 1** suggested MKI67 expression had no effect on the prognosis of BRCA patients and COADREAD patients in TCGA database, while the increased MKI67 expression was significantly associated with poor prognosis of BRCA patients, but with good prognosis of COADREAD patients in our verification results. This may be due to inter-tumor/intra-tumor heterogeneity or the difference in patient population, such as racial or regional disparities. Above all, the prognosis value of MKI67 expression in several cancer types was precisely identified in our validation cohort and TCGA database, of which the results were generally consistent and reliable.

**Table 1:** The comparisons of results between our validation and TCGA database.

Clinical parameters	Verify OS			OS in TCGA database		
	n	Hazard ratio(95%CI)	*p*-Value	n	Hazard ratio(95%CI)	*p*-Value
LIHC	1454	2.039 (1.735-2.397)	<0.001	424	1.639 (1.160-2.315)	0.005
LUADLUSC	3140	1.682 (1.531-1.848)	<0.001	1149	1.226 (1.008-1.492)	0.042
LUAD	2521	1.725 (1.554-1.915)	<0.001	598	1.397 (1.049-1.860)	0.022
LUSC	615	1.212 (0.851-1.728)	0.3165	551	0.881 (0.673-1.153)	0.353
BRCA	2065	2.752 (2.105-3.597)	0.0001	1226	1.122 (0.816-1.542)	0.479
COADREAD	1261	0.726 (0.561-0.940)	0.0136	643	0.815 (0.577-1.152)	0.243
STAD	904	1.127 (0.954-1.331)	0.149	409	0.842 (0.607-1.168)	0.299
CESC	962	0.991 (0.763-1.287)	0.945	309	0.843 (0.530-1.340)	0.467
ESCA	138	1.172 (0.610-2.252)	0.614	174	1.046 (0.646-1.695)	0.853

### 3.4 Correlation analysis between MKI67 expression and immune cell infiltration

**Figure 7:**
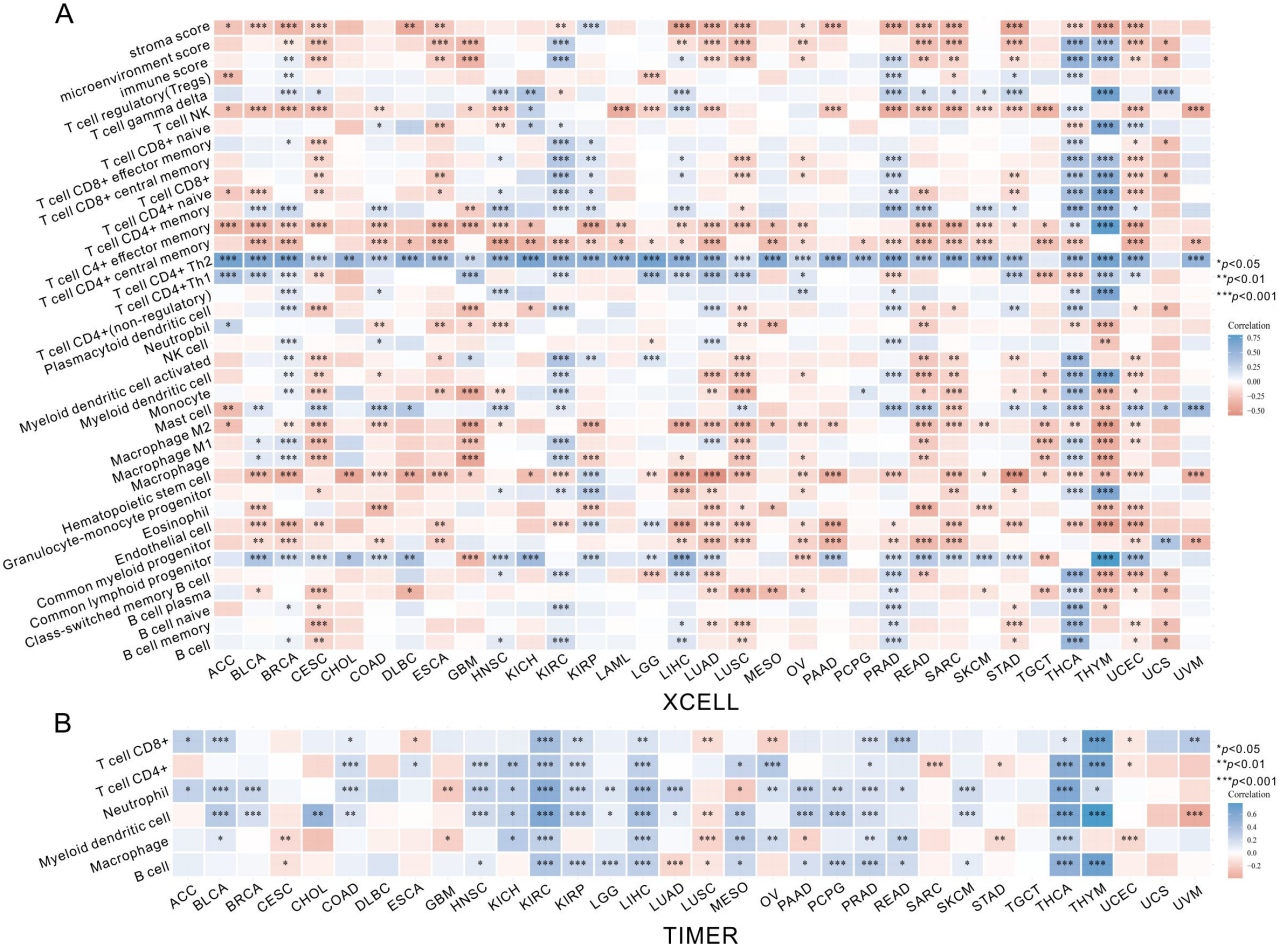
The MKI67 expression correlated with immune infiltration. **(A)** The MKI67 expression significantly correlated with the infiltration levels of various immune cells in the xCell. **(B)** The MKI67 expression significantly correlated with the infiltration levels of various immune cells based on TIMER database. ∗*p* < 0.05, ∗∗*p* < 0.01, and ∗∗∗*p* < 0.001.

After identifying the prognosis value of MKI67 expression, we investigated the potential relationship between MKI67 expression and tumor-infiltrating immune cells, an essential component of the Tumor Microenvironment (TME), across various cancer types by xCell algorithm. As demonstrated in **Figure 7A**, it is revealed that MKI67 expressed in 38 immune cell subtypes generally significantly contributed to the level of tumor-infiltrating immune cells in several cancer types. In particular, MKI67 expression were most positively correlated with Th1 and Th2 CD4^+^ T cells across various cancer types, while MKI67 expression were largely negatively correlated with Macrophage M2 cells across various cancer types. Notably, MKI67 expression have different effect on diverse cancer types, such as positively related to Macrophage M1 cells in BLCA, BRCA, KIRC, LUAD, and THCA; negatively related to Macrophage M1 cells in CESC, GBM, LUSC, READ, TGCT, and THYM. We then verified the relationship between MKI67 expression and tumor-infiltrating immune cells via the TIMER algorithm. **Figure 7B** indicated that the expression of MKI67 was significantly correlated with the tumor purity of 14 cancers and the degree of B-cell invasion of 23 cancers. MKI67 was also associated with CD4^+^ T cell invasion in 22 cancers, CD8^+^ T cell invasion in 19 cancers, DC invasion in 25 cancers, neutrophil invasion in 24 cancers, and macrophage invasion in 17 cancers. Above all, the results revealed that MKI67 expression plays diverse functions in different cancer types, which may partially explain MKI67 performed an opposing impact against the prognosis of various cancer types.

### 3.5 Correlation analysis of MKI67 expression with TMB and MSI

We further explored the relationship between MKI67 expression and dynamic immune-related features, including TMB and MSI. TMB and MSI are two emerging biomarkers related to immunotherapy response. The results showed that MKI67 expression was significantly positively correlated with TMB in various cancer types, including ACC, KICH, STAD, PAAD, BRCA, LUAD, CHOL, and UCS, while it was negatively correlated with TMB in THYM in consistent with prognosis analysis (**Supplementary Figure S1A**). As shown in **Supplementary Figure S1B**, MKI67 expression was positively correlated with MSI in LUSC, STAD, ACC, UCEC, UVM, UCS, and MESO, but negatively correlated with SKCM, PCPG, and DLBC. Moreover, we compared the potential association of MKI67 expression with eight immune checkpoint pathway genes in pan-cancer. The results showed that MKI67 was significantly positively correlated with several of the pan-cancer immune checkpoint genes in some cancer types, such as THCA, STAD, LIHC, and BRCA, but also negatively correlated with the immune checkpoint genes in some cancer types, including THYM and GBM (**Supplementary Figure S1C**). These results revealed that MKI67 was capable of playing a vital role in immunotherapy response.

### 3.6 Enrichment analysis of MKI67 related genes in pan-cancer

Next, we analyzed the MKI67 protein-protein interaction using the String database to further explore the probable molecular mechanisms of it in tumor prognosis (**Supplementary Figure S2**). Then, to investigate the functional impact of the MKI67 gene, we used the GEPIA2 database to extract the top 100 genes with similar expression patterns to MKI67 in all tumor types. Among them, the first 10 genes were positively correlated with MKI67, i.e., IKZF1, DOCK2, NCKAP1L, ARHGAP30, DOCK8, FLI1, VAV1, AKNA, ARHGAP9, and PTPRC, orderly (**Supplementary Figure S3**). We then performed GO and KEGG analyses for these 100 genes. The GO analysis is divided into three parts: GO_MF, GO_BP, and GO_CC (**Supplementary Figure S4A**). Under the GO_BP component, MKI67 was associated with cell proliferation and division in several tumors, such as organelle fission, nuclear Division, and chromosome segregation. For GO_CC, MKI67-related gene products were located in the spindle, chromosomal region, chromosome, and centromeric region and simultaneously perform functions. In the GO_MF analysis, the results indicated that MKI67 related genes mostly had tubulin binding, microtubule binding, and ATP hydrolysis activity. As demonstrated in **Supplementary Figure S4B**, KEGG pathway analysis showed that the 100 genes were mainly related to Cell cycle, Oocyte meiosis, Progesterone mediated oocyte maturation and Human T-cell leukemia virus 1 infection “, “Cellular senescence”, and “p53 signaling pathway”.

## 4 Discussion

Recently, MKI67 has been used more widely against various cancer types in clinical settings as an indicator of cellular proliferation, reflecting proliferative activity of endogenous cell population in malignant tumors (16, 39–41). However, the applicability of MKI67 against certain cancer types was obscure due to different regulatory roles, different prognosis values, and varied cutoff values of MKI67 in diverse cancer types (28, 29, 42–44). Melling et al. report that high MKI67 has a good prognostic value for CRC, associated with low tumor stage and nodal status (45). IKWG Consensus Meeting reports that MKI67 level at 5% or less was significantly associated with good prognosis, while MKI67 level at 30% or more was significantly related to poorer prognosis in ER-positive early-stage breast cancer (32). In this paper, we firstly performed a comprehensive analysis of the prognosis value of Ki67 in various cancer types by 10028 patients collected and several public databases.

Here, we initially observed MKI67 expression significantly increasing across varied cancer types through TIMER2 database, TCGA dataset, and CPTAC dataset, including BLCA, BRCA, CESC, CHOL, COAD, ESCA, GBM, HNSC, KICH, KIRC KIRP. LIHC, LUAD, LUSC, PCPG, PRAD, READ, SKCM, STAD, THCA and UCEC. These results were consistent with previous studies (21). Based on the TCGA database, we found there were radically different MKI67 expressions of stage I, II, III, and IV in LUADLUSC, LIHC, BRCA, and THCA. Additionally, we collected information of 10028 patients with MKI67 expression and corresponding clinical parameters to validate the correlation. We found that high expression of MKI67 was correlated with poor differentiation, TNM classification, and classification in BRCA patients (46–48), and high expression of MKI67 was closely related to BCLA stage, Edmondson grade, tumor size, and tumor nodule increase in LIHC patients (49). Our verification results were almost consistent with the results of other studies and TCGA database. Taken together, there was a close association between the high MKI67 expression and certain clinicopathological features and clinical parameters, such as poor differentiation, TNM classification, and clinical stage, among different cancer types.

To precisely identify the prognosis value in varied cancer types, our study calculated the cutoff value of varied cancer types ROC curves with LUADLUSC at 21.5%, LIHC at 31.5%, BRCA at 36.5%, COADREAD at 66.5%, STAD at 69%, ESCA at 67.5%, and CESC at 74%. We strictly followed up the prognosis of 10028 cancer patients with the top 10 incidence rates in China, including LUNG, COADREAD, THCA, LIHC, STAD, BRCA, ESCA, CESC, PRAD and PAAD patients. Patients we collected and TCGA database displayed an intimate relationship between MKI67 expression level and prognosis across varied cancer types. For example, the low expression group of Ki67 had a longer OS in LUADLUSC, LIHC, and LUAD patients both in our verification results and the TCGA database. The results were consistent with previous studies, which have also found that the median/overall survival of the low Ki67 expression group in LUADLUSC was significantly longer than that of the high Ki67 expression group (50–53). Then, our verification results found that the expression of MKI67 had no effect on the survival of CESC, STAD, and ESCA, which was consistent with the results of the TCGA database. This situation was occasionally reported, such as some studies reported that Ki67 had no prognostic value in gastric cancer, and may only be a potential indicator of intra-tumor heterogeneity (54). Moreover, our verification results suggested that the group with high expression of Ki67 had a longer OS in COADREAD, but MKI67 expression in TCGA database did not affect the survival of COADREAD in TCGA database. However, our findings were consistent with the results of a study involving 1653 CRC patients, in which high expression of Ki67 was associated with good clinical outcomes in CRC patients and with good treatment outcomes in patients receiving adjuvant chemotherapy for colon cancer (45, 55) Notably, our verification results showed that the low expression group of Ki67 had a longer OS, while the expression of MKI67 in the TCGA database did not affect the survival of BRCA. However, our findings are consistent with the study of El Benna H et al., who also found that there was a significant correlation between Ki67 expression and the overall survival of breast cancer patients, which was an independent predictor of patient prognosis and could also predict the effectiveness of chemotherapy or hormone therapy (56, 57). These differences may be due to the lack of standardized methods for quantifying MKI67 expression, inter-tumor/intra-tumor heterogeneity, or the difference in patient population, such as racial or regional disparities (28, 30, 58–60). Above all, MKI67 expression plays distinct and essential roles in the prognosis of various cancer types.

Probing the underlying immune and molecular mechanisms of MKI67 by xCell algorithm, TIMER algorithm, correlation analysis, and enrichment analysis, our results suggested that MKI67 expression was significantly correlated with the level of tumor-infiltrating immune cells across several cancer types, including B cells, CD4^+^ T cells, CD8^+^ T cells, DC infiltrates, neutrophils, and macrophages. Especially, Th1 and Th2 CD4^+^ T cells were most positively correlated with MKI67 expression in various cancer types. The results of TIMER algorithm were slightly different from xCell algorithm, which may be due to the diverse role of immune cells and stromal cells in aggressive malignant progression, including tumor proliferation and invasion, and drug resistance (61). Moreover, we found a significant positive correlation between MKI67 expression and TMB and MSI in different cancer types, including ACC, KICH, STAD, PAAD, BRCA, LUAD, CHOL, and UCS for TMB and LUSC, STAD, ACC, UCEC, UVM, UCS, and MESO for MSI. While a negative correlation between MKI67 expression and TMB and MSI in different cancer types, including THYM for TMB and SKCM, PCPG, and DLBC for MSI (62). The diverse functions of MKI67 expression may partially explicate MKI67 carried out an opposing impact on the prognosis of different cancer types. In addition, the results of enrichment analysis showed that MKI67 was significantly correlated with many signaling pathways, such as p53 signaling pathway and Cell cycle. Some studies have found that p53 inhibits Ki67 promoter activity in a dose-dependent way, and identified the Sp1 binding site responsible for p53-mediated transcription inhibition of Ki67 (63, 64).

In summary, we investigated the expression characteristics, prognostic value, relationship with tumor-infiltrating immune cells, and related pathways of MKI67 in pan-cancer from a multi-group bioinformatics perspective and 10028 patients data in the context of immuno-oncology. It was found that MKI67 may have great potential as a cancer prognostic and immune infiltration marker, whereas the current study was insufficiently validated due to the lack of in-depth experiments to validate its specific immune infiltration relationship with TMEs. In addition, our study was a single-center retrospective study with the disadvantage of a single population type. In the future, multicenter studies can be conducted to expand the population categories and improve the accuracy of the study results.

## Data Availability

Publicly available datasets were analyzed in this study. This data can be found here: TCGA (https://portal.gdc.cancer.gov), Clinical Proteomic Tumor Analysis Consortium (CPTAC) database (https://cptac-dataportal.georgetown.edu/), GEPIA2 database (http://gepia2.cancerpku.cn). In addition, the datasets collected and presented in this study can be found in online repositories. The names of the repository/repositories and accession number(s) can be found in the article/**Supplementary Material**.
