# Supplementary material for: A comprehensive analysis of the prognostic value, expression characteristics and immune correlation of MKI67 in cancers
Source: Front Immunol. 2025 Feb 24;16:1531708. doi: 10.3389/fimmu.2025.1531708 (PMC11894575; doi:10.3389/fimmu.2025.1531708)
Supplement: Supplementary file 1 [file DataSheet1.docx]

**A Comprehensive analysis of the prognostic value, expression characteristics and immune correlation of MKI67 in cancers**

Xiaolan Pan^1#^, Caibiao Wei^1#^, Jingyu Su^2#^, Min Fang^1^ ，Qiumei Lin^1^, Yuling Qin^1^, Jie Gao^1^, Jie Zhao^2*^ ,Huiliu Zhao^1*^, Fengfei Liu^1*^

^1^ Department of Clinical Laboratory, Guangxi Medical University Cancer Hospital, Nanning, China

^2^ Genetic Metabolism Center laboratory, Guangxi Zhuang Autonomous Region Maternal and Child Health Care Hospital, Nanning, China

^3^ Department of Medical Records, Guangxi Medical University Cancer Hospital, Nanning, China

#These authors contributed equally to this work.

*Corresponding Authors: Jie Zhao, E-mail:zhubogxnn@126.com

Huiliu Zhao, E-mail: zhl200308@163.com

Fengfei Liu, E-mail: fengfeiliu_faye@126.com

Patient clinical parameters with MKI67 expression in BRCA. (Table S1) ………………..S 2

Patient clinical parameters with MKI67 expression in LUADLUSC. (Table S2) …………S 3

Patient clinical parameters with MKI67 expression in LIHC. (Table S3)………………….S 4

Patient clinical parameter with MKI67 expression in COADREAD. (Table S4)………….S 5

Patient clinical parameters with MKI67 expression in STAD. (Table S5)………………...S 6

Patient clinical parameters with MKI67 expression in ESCA. (Table S6)………………...S 7

Patient clinical parameters with MKI67 expression in CESC. (Table S7)…………….…..S 8

Correlation analysis of MKI67 expression with TMB and MSI (Fig. S1) ………….…….S9

MKI67 co-expression network (Fig. S2) …………………………………………….….. S10

The correlation of top 10 MKI67-correlated genes (Fig. S3) ……………………….……S10

GO and KEGG analyses for these 100 genes (Fig. S4) ………………………………….S10

Abbreviations……………………………………………………………………………..S11

**Table S1**. Patient clinical parameters and their association with MKI67 expression in BRCA.

| **Clinical parameters** | **n** | **MKI67** | | |
| --- | --- | --- | --- | --- |
|  |  | **Positive** | **Negative** | ***p*-Value** |
| **T classiffcation** |  |  |  | **<0.001** |
| T1 | 631 | 228 | 403 |  |
| T2 | 1144 | 539 | 575 |  |
| T3+T4 | 320 | 172 | 148 |  |
| **N classiffcation** |  |  |  | **<0.001** |
| N0 | 1021 | 403 | 609 |  |
| N1+N2+N3 | 1053 | 536 | 517 |  |
| **M classiffcation** |  |  |  |  |
| M0 | 1967 | 872 | 1095 | **<0.001** |
| M1 | 98 | 67 | 31 |  |
| **Clinical stage** |  |  |  | **<0.001** |
| Ⅰ | 490 | 161 | 329 |  |
| Ⅱ | 1083 | 503 | 580 |  |
| Ⅲ+Ⅳ | 493 | 218 | 275 |  |
| **Molecular classification** |  |  |  | **<0.001** |
| Triple-Negative | 189 | 146 | 43 |  |
| Luminal A | 255 | 13 | 242 |  |
| Lnminal B | 1309 | 585 | 724 |  |
| Her-2 overexpression | 312 | 195 | 117 |  |
| **Differentiated degree** |  |  |  | **<0.001** |
| G1 | 253 | 99 | 154 |  |
| G2 | 948 | 322 | 626 |  |
| G3 | 864 | 518 | 346 |  |
| **Age (years)** |  |  |  | **0.026** |
| ≤60 | 1791 | 832 | 959 |  |
| >60 | 274 | 107 | 167 |  |

**Table S2**. Patient clinical parameters and their association with MKI67 expression in LUADLUSC.

| **Clinical parameters** | **n** | **MKI67** | | |
| --- | --- | --- | --- | --- |
|  |  | **Positive** | **Negative** | ***p*-Value** |
| **T classiffcation** |  |  |  | **<0.001** |
| T1 | 536 | 193 | 343 |  |
| T2 | 731 | 461 | 270 |  |
| T3 | 364 | 271 | 93 |  |
| T4 | 759 | 542 | 217 |  |
| **N classiffcation** |  |  |  | **<0.001** |
| N0 | 606 | 218 | 388 |  |
| N1 | 171 | 119 | 52 |  |
| N2 | 709 | 479 | 230 |  |
| N3 | 883 | 641 | 242 |  |
| **M classiffcation** |  |  |  | **<0.001** |
| M0 | 1139 | 636 | 503 |  |
| M1 | 1200 | 790 | 410 |  |
| **Clinical stage** |  |  |  | **<0.001** |
| Ⅰ | 343 | 90 | 253 |  |
| Ⅱ | 107 | 70 | 37 |  |
| Ⅲ | 463 | 356 | 107 |  |
| Ⅳ | 1166 | 764 | 402 |  |
| **Age (years)** |  |  |  | **0.344** |
| ≤60 | 1474 | 901 | 573 |  |
| >60 | 1660 | 1042 | 618 |  |
| **Gender** |  |  |  | **<0.001** |
| Males | 2025 | 1365 | 660 |  |
| Females | 1109 | 578 | 531 |  |
| **Pathological type** |  |  |  | **<0.001** |
| LUAD | 2519 | 1378 | 1141 |  |
| LUSC | 615 | 565 | 50 |  |

**Table S3**. Patient clinical parameters and their association with MKI67 expression in LIHC.

| **Clinical parameters** | **n** | **MKI67** | | |
| --- | --- | --- | --- | --- |
|  |  | **Positive** | **Negative** | ***p*-Value** |
| **BCLA stage** |  |  |  | **<0.001** |
| A | 687 | 256 | 431 |  |
| B | 228 | 94 | 134 |  |
| C | 302 | 181 | 121 |  |
| **Tumor size(cm)** |  |  |  | **0.002** |
| ≥5 | 626 | 313 | 313 |  |
| <5 | 453 | 184 | 269 |  |
| **Liver cirrhosis** |  |  |  |  |
| Yes | 723 | 332 | 391 | **0.972** |
| No | 731 | 335 | 396 |  |
| **Edmondson grade** |  |  |  | **<0.001** |
| Ⅰ+Ⅱ | 549 | 196 | 380 |  |
| Ⅲ+Ⅳ | 490 | 301 | 189 |  |
| **Node number** |  |  |  | **<0.001** |
| ≥2 | 241 | 125 | 116 |  |
| 1 | 1208 | 538 | 670 |  |
| **MVI** |  |  |  | **<0.001** |
| Negativ | 586 | 198 | 388 |  |
| Positive | 740 | 421 | 319 |  |
| **Age (years)** |  |  |  | **0.001** |
| ≤60 | 1145 | 552 | 593 |  |
| >60 | 309 | 115 | 194 |  |
| **Gender** |  |  |  | **0.572** |
| Males | 1334 | 609 | 725 |  |
| Females | 120 | 58 | 62 |  |
| **Clonorchis sinensis** |  |  |  | **0.842** |
| Yes | 85 | 38 | 47 |  |
| No | 1369 | 629 | 740 |  |

**Table S4**. Patient clinical parameters and their association with MKI67 expression in COADREAD.

| **Clinical parameters** | **n** | **MKI67** | | | | |
| --- | --- | --- | --- | --- | --- | --- |
|  |  | **Positive** | | **Negative** | | ***p*-Value** |
| **T classiffcation** |  |  |  | | **0.086** | |
| T1+ T2 | 277 | 164 | 113 | |  | |
| T3 | 659 | 352 | 307 | |  | |
| T4 | 280 | 140 | 140 | |  | |
| **N classiffcation** |  |  |  | | **0.007** | |
| N0 | 629 | 286 | 343 | |  | |
| N1 | 385 | 214 | 171 | |  | |
| N2 | 200 | 97 | 103 | |  | |
| **M classiffcation** |  |  |  | | **<0.001** | |
| M0 | 1104 | 615 | 489 | |  | |
| M1 | 112 | 41 | 71 | |  | |
| **Clinical stage** |  |  |  | | **0.507** | |
| Ⅰ+Ⅱ | 952 | 520 | 432 | |  | |
| Ⅲ+Ⅳ | 260 | 136 | 124 | |  | |
| **Age (years)** |  |  |  | | **0.162** | |
| ≤60 | 643 | 359 | 284 | |  | |
| >60 | 573 | 297 | 276 | |  | |
| **Gender** |  |  |  | | **0.782** | |
| Males | 731 | 392 | 339 | |  | |
| Females | 485 | 264 | 221 | |  | |
| **Pathological type** |  |  |  | | **0.001** | |
| CR | 624 | 364 | 260 | |  | |
| CC | 589 | 290 | 299 | |  | |
| **Differentiated degree** |  |  |  | | **0.048** | |
| Low | 151 | 81 | 70 | |  | |
| Medium | 964 | 533 | 431 | |  | |
| High | 95 | 40 | 55 | |  | |

**Table S5.** Patient clinical parameters and their association with MKI67 expression in STAD.

| **Clinical parameters** | **n** | **MKI67** | | |
| --- | --- | --- | --- | --- |
|  |  | **Positive** | **Negative** | ***p*-Value** |
| **T classiffcation** |  |  |  | 0.562 |
| T1+T2 | 89 | 40 | 49 |  |
| T3+T4 | 361 | 150 | 211 |  |
| **N classiffcation** |  |  |  | 0.211 |
| N0+N1 | 199 | 77 | 122 |  |
| N2+N3 | 166 | 75 | 91 |  |
| **M classiffcation** |  |  |  | 0.468 |
| M0 | 345 | 145 | 200 |  |
| M1 | 102 | 47 | 55 |  |
| **Clinical stage** |  |  |  | 0.472 |
| Ⅰ+Ⅱ | 69 | 28 | 41 |  |
| Ⅲ+Ⅳ | 193 | 88 | 105 |  |
| **Age (years)** |  |  |  |  |
| ≤60 | 470 | 187 | 283 | <0.001 |
| >60 | 434 | 225 | 209 |  |
| **Gender** |  |  |  | 0.062 |
| Males | 612 | 292 | 320 |  |
| Females | 292 | 120 | 172 |  |

**Table S6.** Patient clinical parameters and their association with MKI67 expression in ESCA.

| **Clinical parameters** | **n** | **MKI67** | | |
| --- | --- | --- | --- | --- |
|  |  | **Positive** | **Negative** | ***p*-Value** |
| **T classiffcation** |  |  |  | 0.945 |
| T1+ T2 | 17 | 9 | 8 |  |
| T3 | 47 | 27 | 20 |  |
| T4 | 20 | 11 | 9 |  |
| **N classiffcation** |  |  |  | 0.571 |
| N0 | 29 | 15 | 14 |  |
| N1+N2+N3 | 55 | 32 | 23 |  |
| **M classiffcation** |  |  |  | 0.135 |
| M0 | 69 | 36 | 33 |  |
| M1 | 15 | 11 | 4 |  |
| **Clinical stage** |  |  |  | 0.917 |
| Ⅰ+Ⅱ | 29 | 16 | 13 |  |
| Ⅲ+Ⅳ | 55 | 31 | 24 |  |
| **Age (years)** |  |  |  | 0.422 |
| ≤60 | 39 | 20 | 19 |  |
| >60 | 45 | 27 | 18 |  |
| **Gender** |  |  |  | 0.928 |
| Males | 71 | 42 | 29 |  |
| Females | 13 | 5 | 8 |  |

**Table S7.** Patient clinical parameters and their association with MKI67 expression in CESC.

| Clinical parameters | **n** | **MKI67** | | |
| --- | --- | --- | --- | --- |
|  |  | **Positive** | **Negative** | ***p*-Value** |
| **Pathological type** |  |  |  | 0.464 |
| Cervical endocervical adenocarcinoma | 727 | 404 | 323 |  |
| Cervical squamous cell carcinoma | 235 | 137 | 98 |  |
| **Clinical stage** |  |  |  | 0.535 |
| Ⅰ | 531 | 290 | 241 |  |
| Ⅱ | 185 | 108 | 77 |  |
| Ⅳ+Ⅲ | 243 | 136 | 98 |  |
| **Age (years)** |  |  |  | <0.001 |
| ≤60 | 525 | 435 | 90 |  |
| >60 | 197 | 106 | 91 |  |
| **Gender** |  |  |  |  |
| Females | 962 | 541 | 421 |  |


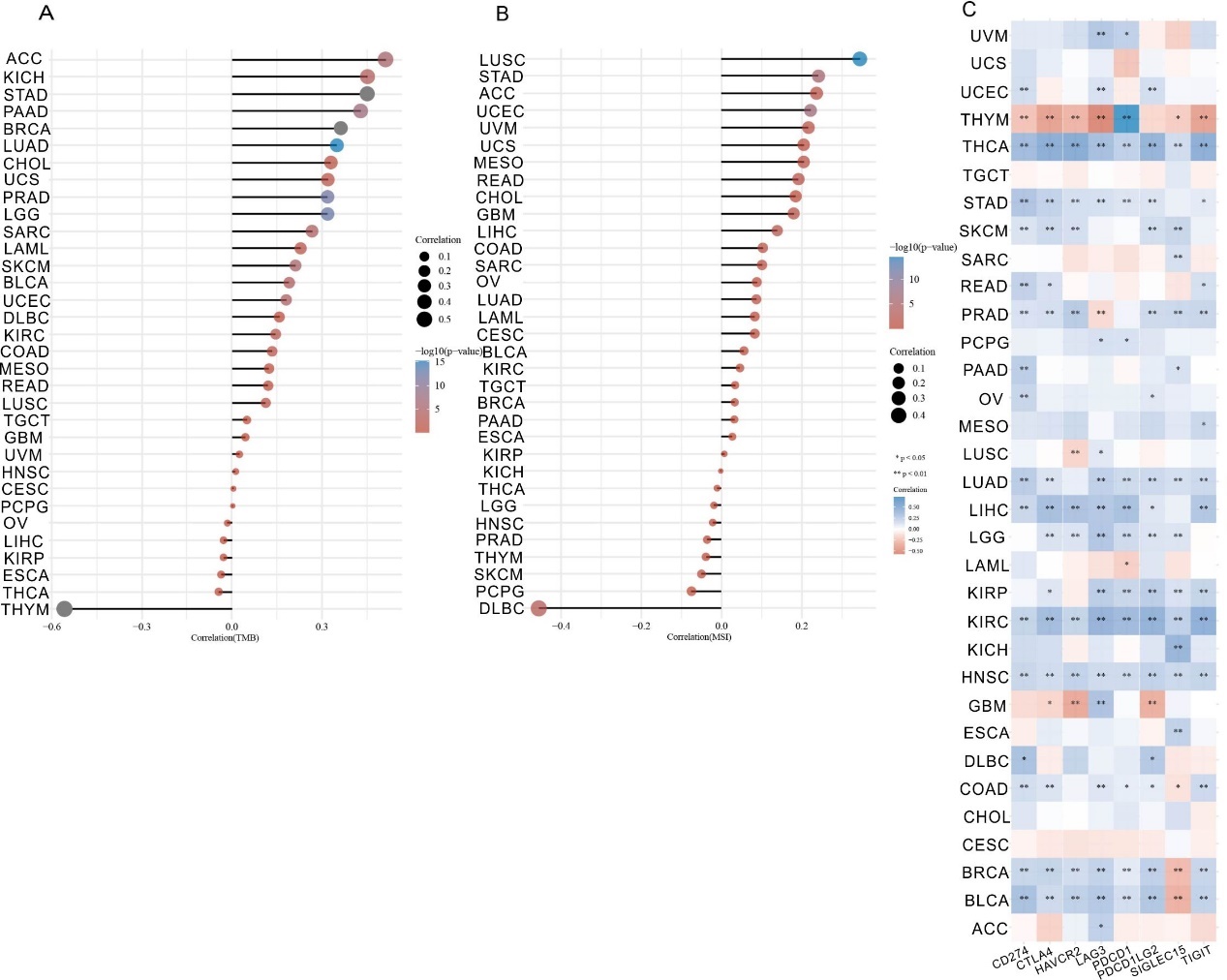


Fig.S1. (A) correlation analyses of the MKI67 expression with immune checkpoint genes in pan-cancer. (B) A stick chart shows the relationship between the MKI67 gene expression and TMB in pan-cancer. (C) A stick chart shows the association between the MKI67 gene expression and MSI in pan-cancer. ∗*p* < 0.05, ∗∗*p* < 0.01, ∗∗∗*p* < 0.001, and ∗∗∗∗*p* < 0.0001.


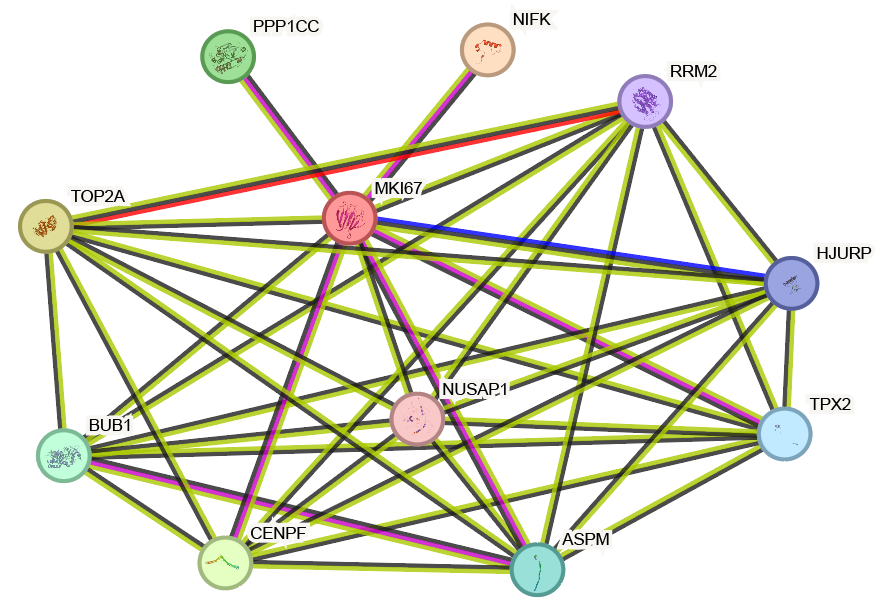


**Fig S2**. MKI67 co-expression network.


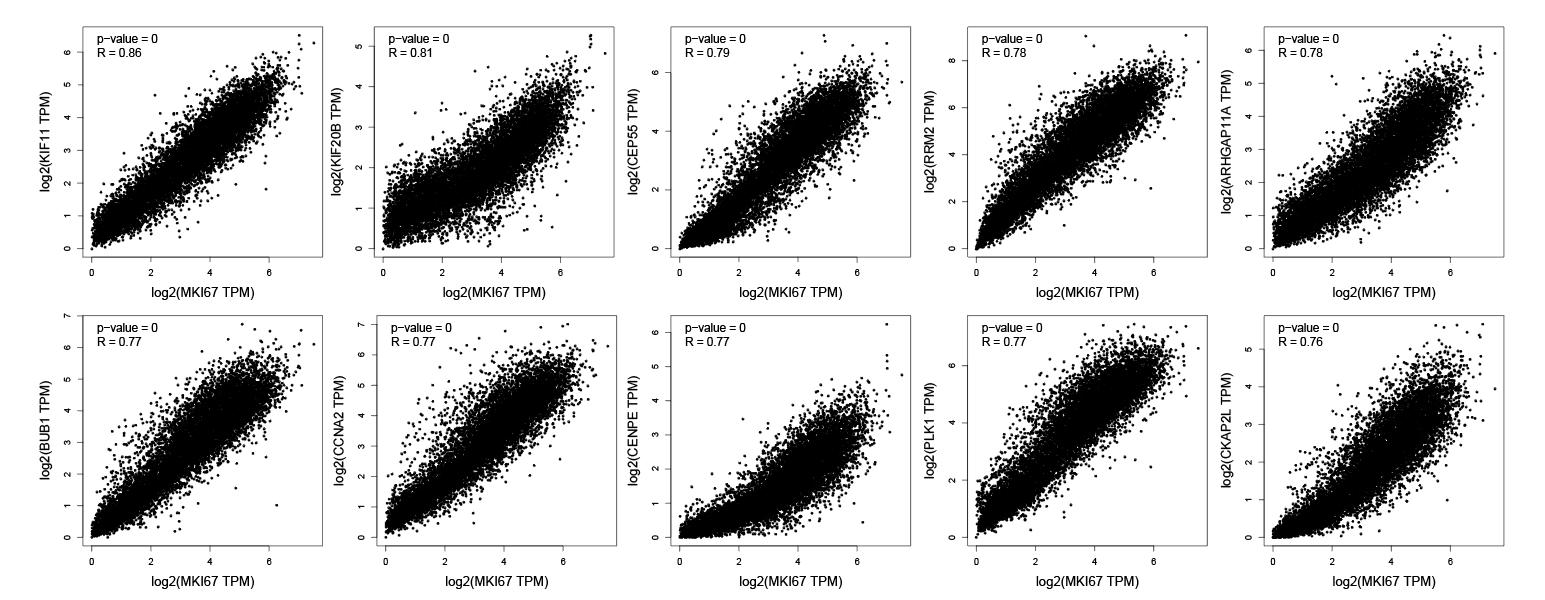


**Fig S3.** The correlation of top 10 MKI67-correlated genes, including KIF11, KIF20B, CEP55, RRM2, ARHGAP11A, BUB1, CCNA2, CENPE, PLK and CKAP2L, in order.


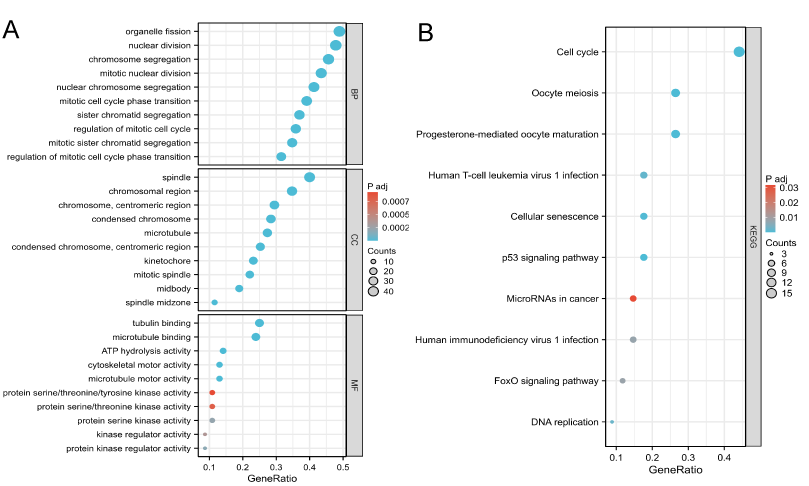


**Fig S4.** (A) GO_BP analysis, GO_CC analysis and GO_MF analysis. (B) KEGG pathway analysis.

**Abbreviations**

| MKI67 | Marker of proliferation Ki-67 |
| --- | --- |
| ACC | Adrenocortical carcinoma |
| BLCA | Bladder Urothelial Carcinoma |
| CESC | Cervical squamous cell carcinoma and endocervical adenocarcinoma |
| CHOL | Cholangiocarcinoma |
| COAD | Colon adenocarcinoma |
| DLBC | Lymphoid Neoplasm Diffuse Large B-cell Lymphoma |
| GBM | Glioblastoma multiforme |
| HNSC | Head and Neck squamous cell carcinoma |
| KICH | Kidney Chromophobe |
| KIRC | Kidney renal clear cell carcinoma |
| KIRP | Kidney renal papillary cell carcinoma |
| LGG | Brain Lower Grade Glioma |
| LUAD | Lung adenocarcinoma |
| LUSC | Lung squamous cell carcinoma |
| MESO | Mesothelioma |
| OV | Ovarian serous cystadenocarcinoma |
| PCPG | Pheochromocytoma and Paraganglioma |
| READ | Rectum adenocarcinoma |
| SKCM | Skin Cutaneous Melanoma |
| STAD | Stomach adenocarcinoma |
| THYM | Thymoma |
| UCEC | Uterine Corpus Endometrial Carcinoma |
| UCS | Uterine Carcinosarcoma |
| UVM | Uveal Melanoma |
